# Skeletal Muscle Oxygen Saturation (StO_2_) Measured by Near-Infrared Spectroscopy in the Critically Ill Patients

**DOI:** 10.1155/2013/502194

**Published:** 2013-08-21

**Authors:** J. Mesquida, G. Gruartmoner, C. Espinal

**Affiliations:** Critical Care Department, Hospital de Sabadell, Corporació Sanitària Universitària Parc Taulí, Universitat Autònoma de Barcelona, 08208 Sabadell, Spain

## Abstract

According to current critical care management guidelines, the overall hemodynamic optimization process seeks to restore macrocirculatory oxygenation, pressure, and flow variables. However, there is increasing evidence demonstrating that, despite normalization of these global parameters, microcirculatory and regional perfusion alterations might occur, and persistence of these alterations has been associated with worse prognosis. Such observations have led to great interest in testing new technologies capable of evaluating the microcirculation. Near-infrared spectroscopy (NIRS) measures tissue oxygen saturation (StO_2_) and has been proposed as a noninvasive system for monitoring regional circulation. The present review aims to summarize the existing evidence on NIRS and its potential clinical utility in different scenarios of critically ill patients.

## 1. Introduction

Tissue hypoxia, as results of oxygen supply-demand imbalance at the cellular level, defines circulatory insufficiency or shock. Maintained over time, this situation might lead to cellular and organ dysfunction, organ damage, and the ultimate death of the individual. In our daily clinical practice, hemodynamic resuscitation of shock states aims to restore global tissue oxygenation markers, such as venous saturations (either central or mixed) or lactate. Including these endpoint variables in the management of shock has led to remarkable improvements in the survival of critically ill patients [1]. However, there is overwhelming evidence demonstrating that, despite normalization of these global tissue hypoxia markers, perfusion disorders might persist at the microcirculatory level [2, 3]. Importantly, persistence of these alterations has been independently associated with further development of multiple organ failure (MOF) and poor outcome [4, 5]. Consequently, over the last years there has been growing interest in developing new technologies capable of assessing the regional circulation and/or the microcirculation [6, 7].

Evaluating the microcirculation in the critically ill patients has been associated with more than a few technical problems, which have delayed their use at the bedside. In addition to the technical limitations, a clinically relevant issue has been finding the right place to monitor. Since any used technology can only assess the microvascular bed of a given sampled tissue, it is necessary to choose accessible territories and yet sufficiently representative of the whole body wellness. Currently, there are several technologies available for the evaluation of the microcirculation [6], which can be classified into two main groups: (a) firstly, direct methods, which allow the visualization of the microvascular bed (such as videomicroscopy); and (b) secondly, indirect methods based on measures of tissue oxygenation, as surrogates of microcirculatory perfusion. In the latter group we can include technologies such as gastric tonometry, tissue oxygen electrodes, sublingual capnometry, and near-infrared spectroscopy (NIRS). Due to its noninvasive nature and its easy applicability, NIRS technology has aroused increasing interest in the evaluation of the regional circulation. This review aims to summarize what, today, has shown this technology in the field of the critically ill patients.

## 2. Near-Infrared Spectroscopy (*NIRS*): Basic Principles

The concept of NIRS technology has already been available for many decades, and it has been developed for different purposes, ranging from chemical analysis in agriculture to pharmaceutical and medical applications. In the late seventies, first noninvasive NIRS devices were used to monitor cerebral and myocardial oxygenation status in living tissues [8], suggesting that the NIRS spectrum of light was perfectly suited for monitoring *in vivo* tissue oxygen provision and utilization. Since then, many studies in humans, along with the development of portable, noninvasive NIRS systems, account for the growing interest in this technology [9, 10].

NIRS technology is based on measuring the attenuation of light in the near-infrared spectrum (700–1000 nm wavelengths) to measure the chromophores, mainly hemoglobin, present in the sampled tissue. Although many other chromophores can influence the obtained NIRS signal (such as bilirubin, melanin, myoglobin, and cytochrome a,a3), choosing specific wavelengths allows for minimizing the impact of these substances, and the obtained signal is derived mainly from oxy- and deoxyhemoglobin. The equipment required for an NIRS system consists of a light source, optical bundles (optodes) for light emission and reception, a processor, and a display system [9]. The distance between the point of light entry and exit (optode separation) will determine the magnitude of sampled tissue. The NIRS signal is derived mainly from the hemoglobin contained in the entire vascular tree and mainly small vessels (arterioles, capillaries, and venules) present in the sampled area [9–13]. Finally, oxy- and deoxyhemoglobin measures permit calculating the overall saturation for tissue hemoglobin or tissue oxygen saturation (StO_2_) [13]. NIRS systems can also provide an estimation of the amount of hemoglobin contained in the sampled area, displayed as total tissue hemoglobin (HbT) or absolute tissue hemoglobin index (THI).

Although StO_2_ has been evaluated in several organs (brain, kidney, and liver), for resuscitation purposes, skeletal muscle StO_2_, due to its nonvital peripheral organ condition, has emerged as a potential early detector of occult hypoperfusion. This review will focus on the usefulness of StO_2_ derived from skeletal muscle in the critical patient. Several muscle locations have been used in the critical care setting. Since StO_2_ measurements derived from the NIRS signal might be altered by local factors such as edema and adipose tissue thickness, some authors have proposed the thenar eminence as a reliable site, less subject to inter- and intraindividual variabilities [13, 14]. Although the thenar eminence is the most widely tested area, interesting results have been obtained also when measuring StO_2_ on muscle locations, such as masseter, deltoid, and the knee area [15, 16]. In healthy basal conditions, the NIRS signal reflects predominantly the venous oxygenation, since an estimated 75% of the blood present in the skeletal muscle is located in the venous compartment [9]. In 700 healthy volunteers, the baseline StO_2_ value measured in the thenar eminence was 87% ± 6% [17]. Similar to mixed venous oxygen saturation, StO_2_ reflects the balance between local oxygen supply and consumption, and any measured change in StO_2_ could be interpreted in both directions: changes in local microcirculatory flow and/or changes in local consumption. Moreover, inversely proportional changes in local flow and consumption could lead to relatively stable values of StO_2_ [6].

## 3. Vascular Occlusion Test (VOT)

**Figure 1:**
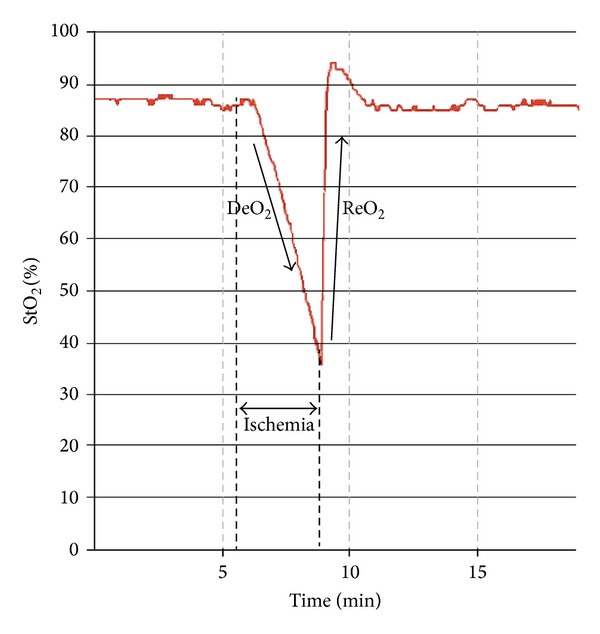
StO_2_ response to a vascular occlusion test (VOT). The transient ischemia generates two main parameters: the deoxygenation response (DeO_2_) and the reoxygenation response (ReO_2_). StO_2_: tissue oxygen saturation; DeO_2_: deoxygenation slope; ReO_2_: reoxygenation slope.

In addition to monitoring the absolute StO_2_ value in the thenar eminence, the StO_2_ response to a brief ischemic challenge has been explored, in order to obtain dynamic information on tissue performance. The so-called vascular occlusion test (VOT) consists in executing an arterial occlusion, proximal to the StO_2_ probe (usually by means of a tourniquet system on the forearm), until a given ischemic threshold is reached, and then the occlusion is released. This test allows generating some dynamic parameters and specially the initial Hb deoxygenation slope (or DeO_2_; expressed as % over time) in the phase of ischemia, followed by the Hb reoxygenation slope (or ReO_2_; also expressed in % over time) once the vascular occlusion is released (Figure 1).

Since DeO_2_ represents the progressive Hb desaturation in a zero-flow situation, it has been proposed as a marker of local oxygen extraction. Correcting the DeO_2_ slope for the estimated local amount of Hb derives a parameter of local oxygen consumption, expressed as nirVO_2_, as proposed by Skarda et al. [21]: nirVO_2_ = (DeO_2_)^−1^/[ (THI_start_ + THI_end_)/2]. On the other hand, ReO_2_ reflects the Hb resaturation, and this will directly depend on blood inflow and capillary recruitment after the hypoxic stimulus. ReO_2_ has been named as a reflection of endothelial function; however, several observations have also correlated ReO_2_ to perfusion pressure [23, 24], and, thus, the resulting ReO_2_ seems to be derived from the interaction of perfusion pressure and endothelial integrity. In its recovery, absolute StO_2_ may temporarily raise the above previous baseline values, indicating postischemic vasodilation and capillary recruitment, also known as reactive hyperemia (Figure 1).

Different VOT methodologies have been described, some of them aimed at maintaining a fixed time of ischemia (3–5 minutes), and some of them sought for an ischemic threshold (StO_2_ drops until a specific value). The lack of standardization of the VOT has led to great difficulties when trying to compare results from different studies. This fundamental issue represents an important limitation of the test, along with the variety of sampled depths and sites used to measure the StO_2_ response to ischemia [14, 25]. According to the existing literature [14, 25], maintaining the ischemic stimulus until a determined StO_2_ value is achieved seems to minimize inter-individual variations, thus homogenizing ReO_2_ values for their comparison. Future consensus should also be applied to the location and depth of measurement of StO_2_ [14].

## 4. StO_2_ in the Critically Ill Patients

While the NIRS technology was developed several decades ago, the new noninvasive and portable NIRS systems emerged as an attractive technology for early detection of shock states in armed conflicts. Thus, initial efforts addressed mainly the value of these systems in hypovolemic shock. After some promising results, NIRS was also explored in other critical conditions and particularly in septic shock. 

### 4.1. StO_2_ in Hypovolemic Shock

In low blood flow states secondary to hypovolemia (such as hemorrhagic shock) the activation of the sympathetic nervous system causes blood flow redistribution from the periphery to the central compartment, through vasoconstriction in certain territories, in order to maintain an optimal perfusion of vital organs [26]. This compensatory mechanism can mask significant hypovolemia associated with hypoperfusion in certain territories, with significant negative impact on outcome [1]. Accordingly, in situations of hypovolemia, a decrease in blood flow to skeletal muscle is expected, with increases in oxygen extraction and decreases in the content of hemoglobin at the regional level. Thus, hypothetically, the evaluation of peripheral perfusion by using StO_2_ seems highly interesting as an early marker of tissue hypoperfusion caused by hypovolemia [27].

This hypothesis was initially tested in experimental conditions, in animal models of hemorrhagic shock. First observations correlated StO_2_ to global variables of flow and oxygen delivery [28–30], suggesting that regional oxygenation measured by NIRS would be able to noninvasively detect progressive hypovolemia. Crookes et al. [31], in a prospective model of resuscitation from hemorrhagic shock, concluded that StO_2_ was a better discriminator for survivors to hemorrhage than mixed venous oxygen saturation (SvO_2_), blood lactate, and base deficit. In human models of central hypovolemia in healthy subjects, StO_2_ and the tissue hemoglobin index (THI) fall have been shown to detect decreases in blood volume equivalent to 400–500 cc, even before the onset of tachycardia or hypotension [32–34]. In addition to its ability to detect progressive hypovolemia, StO_2_ has also been tested for its utility in guiding intravascular volume optimization. On that behalf, Cohn et al., in a prospective randomized pilot study in patients undergoing elective colorectal surgery, analyzed the impact of a standard versus restrictive fluid approach on tissue oxygenation and development of complications [35]. The authors concluded that the restrictive approach was not associated with lower StO_2_ values, suggesting that StO_2_ would be a useful parameter for guiding fluid administration during surgery, ensuring tissue wellness, and avoiding unnecessary fluid overload, which has repeatedly been associated to higher morbidity derived from surgery [36, 37].

In trauma patients, StO_2_ correlation to parameters of flow and oxygen delivery has been also verified [38]. Furthermore, the absolute value of StO_2_ has repeatedly demonstrated its prognostic value in this patient population. Low StO_2_ values during the initial approach to these patients have been associated with larger transfusion requirements [39–41], increased risk of infection [42], multiorgan failure [42, 43], and even higher mortality rates [43, 44]. Importantly, this predictive value was maintained in apparently stable hemodynamic conditions (defined as systolic blood pressure > 90 mmHg) [40, 41]. In addition to absolute StO_2_ values, dynamic variables derived from the VOT have also shown their prognosis in trauma patients [45, 46]. In a recent publication, Guyette et al. [45] demonstrated that early alterations in DeO_2_ were independently associated with the need for early interventions (red blood cell transfusion, emergent surgery, etc.). In this observational study, DeO_2_ was superior to absolute StO_2_ for predictive purposes. Once again, this association was independent and more sensitive than other physiological variables, such as heart rate or blood pressure. Despite the cumulative evidence on the prognostic value of StO_2_ in trauma, with its potential use for early identification of at-risk patients, to this day, there is a lack of prospective studies exploring the usefulness of StO_2_ in trauma resuscitation algorithms, either as a trigger for interventions or as a target in the hemodynamic resuscitation process.

### 4.2. StO_2_ in Severe Sepsis and Septic Shock

The value of StO_2_ has been also widely explored in patients with severe sepsis and septic shock. While absolute StO_2_ values have shown robust prognostic implications in trauma patients, in septic conditions this association appears to be more complex [15, 47, 48]. Although septic patients tend to show lower StO_2_ values than healthy subjects, there is a huge overlap between these populations [19]. These observations could be derived from the heterogeneous nature of microcirculatory alterations in sepsis (ischemic and highly oxygenated coexisting areas), with an overall “normal” oxygen content in a given sensed area [27]. The low sensitivity for these conditions would be a major limitation of absolute StO_2_. Nevertheless, dynamic StO_2_ VOT-derived variables have yielded much more promising results than the absolute StO_2_ in terms of prognosis.

Several authors have reported alterations in the StO_2_ response to the VOT in sepsis, and the magnitude of such alteration has been directly correlated with the development of organ failure, ICU length of stay, or even mortality [15, 18, 20, 23, 49] (Table 1). Alterations in DeO_2_, represented by lower deoxygenation rates, have been associated with poor prognosis. Since DeO_2_ reflects local oxygen consumption, it seems reasonable to hypothesize that patients with limited oxygen extraction will develop higher degrees of organ failure [18, 23]. This local oxygen consumption limitation may be due to two different but cumulative mechanisms: (a) a local supply-demand dependency on low flow or inadequate flow conditions or (b) a low oxygen extraction at the cellular level due to mitochondrial dysfunction and/or alteration of oxygen diffusion (interstitial edema) [23, 50]. Regrettably, the NIRS technology is unable to determine which of these two mechanisms presents greater contribution to the final DeO_2_. Regarding the ReO_2_ slope, it is also diminished in septic patients when compared to healthy subjects [19, 20, 22, 23, 48]. Moreover, the magnitude of ReO_2_ decreased slope has also been correlated to the severity of the episode, and some studies have even demonstrated association with mortality [19, 22, 48]. Not only the initial ReO_2_ value but the persistence of alterations in ReO_2_ during resuscitation has been associated with worse prognosis [19].

## 5. Adding StO_2_ to Current Resuscitation Algorithms?

Although, as we have exposed, StO_2_ has consistently demonstrated its prognostic value in critically ill patients, there is still so much to explore in terms of its clinical applicability at the bedside. One of the major issues that needs to be faced is where to incorporate StO_2_ in hemodynamic resuscitation algorithms and, of course, testing whether StO_2_ incorporation is associated with improvement in outcomes.

### 5.1. Early

Due to its condition of noninvasive continuous measurement of regional oxygenation status, StO_2_ was initially explored in its ability to early detect hypoperfusion, and previously to monitor parameters that require invasive procedures or laboratory analysis. Some authors explored the correlation of StO_2_ with parameters of global oxygenation, such as central venous oxygen saturation (ScvO_2_) [51–55], demonstrating that low StO_2_ values (i.e., StO_2_ < 75% when measured on the thenar eminence) specifically predict extremely low ScvO_2_ values [15, 51, 52]. However, the sensitivity of StO_2_ variables to detect these situations of global hypoperfusion is considerably low, and therefore the absolute StO_2_ value has been proposed as an initial tool to rapidly and noninvasively detect hypoperfusion states, but only while other more sensitive variables are not available [23, 51, 52]. In conclusion, in situations of apparent hemodynamic stability in which we do not have invasive oxygenation parameters, NIRS-derived variables might be useful in the detection of at-risk patients, justifying the need for the beginning of the reanimation process, as well as a more aggressive monitoring [45, 51].

### 5.2. Late

Cumulative evidence on the association between microcirculatory alterations persistence, despite normalization of macrohemodynamic variables, and poor prognosis [1] has led to the idea that evaluating regional oxygenation parameters should be performed at the end of conventional “global” resuscitation. In addition to several *in vivo* videomicroscopy studies, Lima et al. recently found, in a population of septic patients, that alterations in StO_2_ values at the end of the Early Goal-Directed Therapy (EGDT) were associated with higher degrees of organ failure and mortality [16]. Some other studies have also shown consistent data regarding StO_2_ parameters and prognosis despite macrohemodynamic normalization [16, 23]. Unfortunately and once again, there is a lack of prospective interventional studies analyzing the usefulness of adding StO_2_ to the resuscitation algorithm. The fact that whether StO_2_ might be used instead of or complementary to current global tissue oxygenation endpoints, such as ScvO_2_ and lactate, deserves further investigation. In a small pilot study, Nardi et al. [56] attempted to incorporate the StO_2_ measured at three different locations as an endpoint parameter for resuscitation in septic patients. In their protocol, once ScvO_2_ values were normalized according to the Surviving Sepsis Campaign guidelines, StO_2_ goals were pursued in the treatment group. The authors found no benefit in the inclusion of StO_2_ in the resuscitation algorithm. However, the small sample size, the use of the absolute value of StO_2_ instead of dynamic parameters (DeO_2_ or ReO_2_), and the fact that a large percentage of patients in the treatment group did not even normalize the endpoint ScvO_2_ values might account for the lack of differences observed in the evolution of the patients.

One might conclude that prospective studies are needed to evaluate the usefulness of adding StO_2_ to our current macrohemodynamic approach to resuscitation. This major limitation would be applied, to date, to every single regional or microcirculatory monitoring system. 

## 6. Further StO_2_ Applications in Intensive Care

In addition to its potential application in shock states, the StO_2_ may have utility in other clinical scenarios in critical care. Continuous StO_2_ monitoring has shown encouraging results in cardiovascular performance challenges, as in weaning from mechanical ventilation [57]. In a recent study, our group noted that changes in DeO_2_ within a 30-minute spontaneous breathing trial discriminated patients who would succeed in from those who would fail the disconnection from mechanical ventilation process [57], supporting the role of StO_2_ as a monitoring system for detecting limited cardiovascular reserve.

## 7. Technology Limitations

Several limiting factors deserve mention, as they might interfere in StO_2_ values and/or interpretation [58, 59]: (a) exogenous factors, such as changes in environment temperature; (b) endogenous factors such as age, obesity, body temperature, tissue edema, vascular diseases, and agitation; (c) drugs that modify vascular tone [24]. We already commented on the fact that the heterogeneous nature of microcirculatory alterations in septic shock might limit the value of some of the data obtained using the NIRS technology [27]. Finally, it is important to account for an important consideration about this technique: NIRS is a relatively new technology for monitoring the regional circulation in critical care, where no “gold standard” has been validated. However, instead of representing a limitation, the latter might stand for an “everything needs to be done” in regional perfusion and microcirculation in the critically ill patients.

## 8. Conclusions 

In conclusion, StO_2_ and its dynamic variables derived from the VOT have demonstrated their prognostic value in several critical scenarios. The lack of randomized controlled trials analyzing their inclusion in the resuscitation process is the main constraint to the use of this technology at the present time. In addition to its potential value in resuscitation, StO_2_ variables might be useful in other clinical settings, where cardiovascular performance needs to be challenged, such as weaning from mechanical ventilation.

## Figures and Tables

**Table 1:** Summarized prognostic studies on StO_2_ with VOT-derived parameters in septic patients.

Study	Patient population (*n*)	Inclusion time	StO_2_ site/depth	VOT	MAP (mmHg)	DeO_2_ (%/min)	ReO_2_ (%/sec)	Mortality	Comments
Parežnik et al. [18]	SS (6) and SShock (6)	First 48 h (after stabilization)	TH 15 mm	StO_2_ 40%	—	SS −10.4 (−7.8, −13.3) SShock −7 (−3.6, −11)	—	No correlation to StO_2_-derived variables	DeO_2_ correlated to SOFA score

Creteur et al. [19]	SS and SShock (72)	First 24 h	TH 25 mm	3 min	72 (67–79)	—	SShock 2 (1.2, 2.9) versus no SShock 3.2 (1.8, 4.5) (*P* < 0.05)	ReO_2_ correlated to mortality SV 3.2 ± 1.3 NonSV 1.9 ± 1.3 (*P* < 0.05)	AUC 0.797 ReO_2_ cut-off 2.55 (S 85%, E 73%)

Doerschug et al. [20]	Sepsis (24)	First 24 h	TH 15 mm	5 min	69 (max 90, min 55)	—	Moderate sepsis 3.6 ± 1.2 Severe sepsis 2.3 ± 1.5	ReO_2_ tended to be higher in SV than in NonSV 3.3 ± 1.4 versus 2.5 ± 1.5 (*P* 0.2)	

Skarda et al. [21]	SS and SShock (10)	ICU admission	TH 15 mm	3 min	73 ± 11	−11.2 ± 2.4	2.3 ± 1.0	No association between StO_2_ variables and mortality	

Payen et al. [22]	SShock (43)	First 24 h (after vasopressors)	TH 25 mm	3 min	75 (65–82)	−18.6 (−28.2, −14.4)	2.79 (1.75, 4.52)	ReO_2_ correlated to mortality SV 3.9 (2.2, 6.0) NonSV 1.9 (1.6, 2.8) (*P* 0.003)	AUC 0.77 ReO_2_ cut-off 2.83 (S 87%, E 67%)

Mesquida et al. [23]	SShock (33)	First 24 h, once MAP > 65 mmHg	TH 15 mm	StO_2_ 40%	79 ± 12	−12.2 ± 4.2 SOFAimp −13.8 ± 4.3 SOFAnonimp −9.8 ± 2.9	3.02 ± 1.7	DeO_2_ tended to be lower in NonSV than in SV (*P* ns)	DeO_2_ associated with SOFA evolution and ICU-LOS ReO_2_ associated with ICU-LOS

StO_2_: tissue oxygen saturation; VOT: vascular occlusion test; DeO_2_: StO_2_-deoxygenation slope; ReO_2_: StO_2_-reoxygenation slope; SS: severe sepsis; SShock: septic shock; TH: thenar; SOFA: sequential organ failure assessment; SV: survivors; NonSV: nonsurvivors; AUC: area under the curve; SOFAimp: SOFA improvers at day 2; SOFAnonimp: SOFA nonimprovers at day 2; LOS: length of stay.
